# Action information contributes to metacognitive decision-making

**DOI:** 10.1038/s41598-020-60382-y

**Published:** 2020-02-27

**Authors:** Martijn E. Wokke, Dalila Achoui, Axel Cleeremans

**Affiliations:** 1 Programs in Psychology and Biology, The Graduate Center of the City University of New York, New York, NY USA; 2 Department of Psychology, The University of Cambridge, Cambridge, UK; 3 Consciousness, Cognition, and Computation Group, Université Libre de Bruxelles, 1050 Bruxelles, Belgium; 4 Center for Research in Cognition and Neurosciences, Université Libre de Bruxelles, 1050 Bruxelles, Belgium; 5 Neuroscience Institute, Université Libre de Bruxelles, 1050 Bruxelles, Belgium

**Keywords:** Decision, Human behaviour

## Abstract

Metacognitive abilities allow us to adjust ongoing behavior and modify future decisions in the absence of external feedback. Although metacognition is critical in many daily life settings, it remains unclear what information is actually being monitored and what kind of information is being used for metacognitive decisions. In the present study, we investigated whether response information connected to perceptual events contribute to metacognitive decision-making. Therefore, we recorded EEG signals during a perceptual color discrimination task while participants were asked to provide an estimate about the quality of their decision on each trial. Critically, the moment participants provided their confidence judgments varied across conditions, thereby changing the amount of action information (e.g., response competition or response fluency) available for metacognitive decisions. Results from three experiments demonstrate that metacognitive performance improved when first-order action information was available at the moment metacognitive decisions about the perceptual task had to be provided. This behavioral effect was accompanied by enhanced functional connectivity (beta phase synchrony) between motor areas and prefrontal regions, exclusively observed during metacognitive decision-making. Our findings demonstrate that action information contributes to metacognitive decision-making, thereby painting a picture of metacognition as a process that integrates sensory evidence and information about our interactions with the world.

## Introduction

The ability to monitor and evaluate the quality of our decision-making is crucial for adept behavior. For instance, when driving a car for a long time it is important to have a reliable estimate about the adequacy of one’s driving performance to avoid unsafe situations. However, not much is known how our brain constructs such an estimate, or what exactly is being monitored and evaluated. In lab settings, perceptual or memory tasks have been frequently used to probe the mechanisms that underpin metacognitive performance^1–3^. In such studies, first-order task performance generally correlates with second-order (metacognitive) decisions, leading to the intuitive assumption that metacognitive decisions are largely based on the same information that governs first-order decision-making^4–6^.

In recent years, however, dissociations between objective task performance and subjective ratings, and dissociations between sources of information supporting first- and second-order decisions have been observed^7–12^. Typically, metacognitive decisions are provided after first-order responses, thereby allowing certain sources of information to become available during second-order decision-making. Recent findings suggest that metacognition can be supported by ‘embodied’ processes, such as interoception or response information that become available for metacognitive decision-making after a first-order decision has been made^7,13–16^. For instance, manipulation of neural activity via transcranial magnetic stimulation over premotor cortex resulted in altered confidence judgments during a perceptual task^8^. Critically, stimulation of premotor areas reduced metacognitive capacity without changing visual discrimination performance. Further, it has been shown that the order of rating confidence (before or after the response) influenced metacognitive performance on an anagram problem-solving task^17^. From a computational perspective, Pasquali and colleagues explored neural network architectures aimed at capturing the complex relationships between first-order and second-order (metacognitive) performance in a range of different cognitive tasks and suggested that metacognitive judgments are rooted in learned redescriptions of first-order error information rather than in the relevant first-order information itself^18^. This is broadly consistent with Fleming and Daw’s perspective, in which they offered to unify the above observations in a single framework in which confidence operates as a second-order computation about one’s own performance^19^. In this framework, samples of sensory evidence that support first- and second-order decisions are coupled yet distinct. Interestingly, their second-order model of confidence computation incorporates knowledge about the reliability of actions towards perceptual events.

Here, in three experiments, we aimed to elucidate whether and in what way action information informs metacognitive judgments. We therefore constructed a color discrimination task in which we varied the amount of available action information (i.e., response strength and fluency of response execution) at the moment a metacognitive judgment had to be provided. Our design enabled us to contrast metacognitive decisions based on purely perceptual information (uninformed by action processes) with metacognitive decisions having access to both perceptual and motor action information. We recorded electroencephalographic signals to investigate whether functional connectivity between motor regions and prefrontal cortex could serve as a mechanism to convey relevant action information (e.g., response competition or response fluency) during metacognitive decision-making.

Previously, beta oscillations have been intimately linked to sensory and motor processing^20^. Recently, however, beta-band power (de)synchronization in motor regions has been shown to provide insight into the dynamics underlying perceptual decisions^21^ and response uncertainty^22^. Beta oscillations have repeatedly been shown to predict first-order decisions^22–24^, to support maintenance of persistent activity^25–27^ to mediate long-range communication, and to play an important role in the preservation and ‘awakening’ of endogenous information^28^. Here, we focused on beta phase synchrony between motor regions and prefrontal cortex^9^. Specifically, we expected both functional connectivity (beta phase synchrony) and metacognitive performance to increase when response information about first-order decisions would be accessible during metacognitive decision-making.

## Results

### Behavior

**Figure 1:**
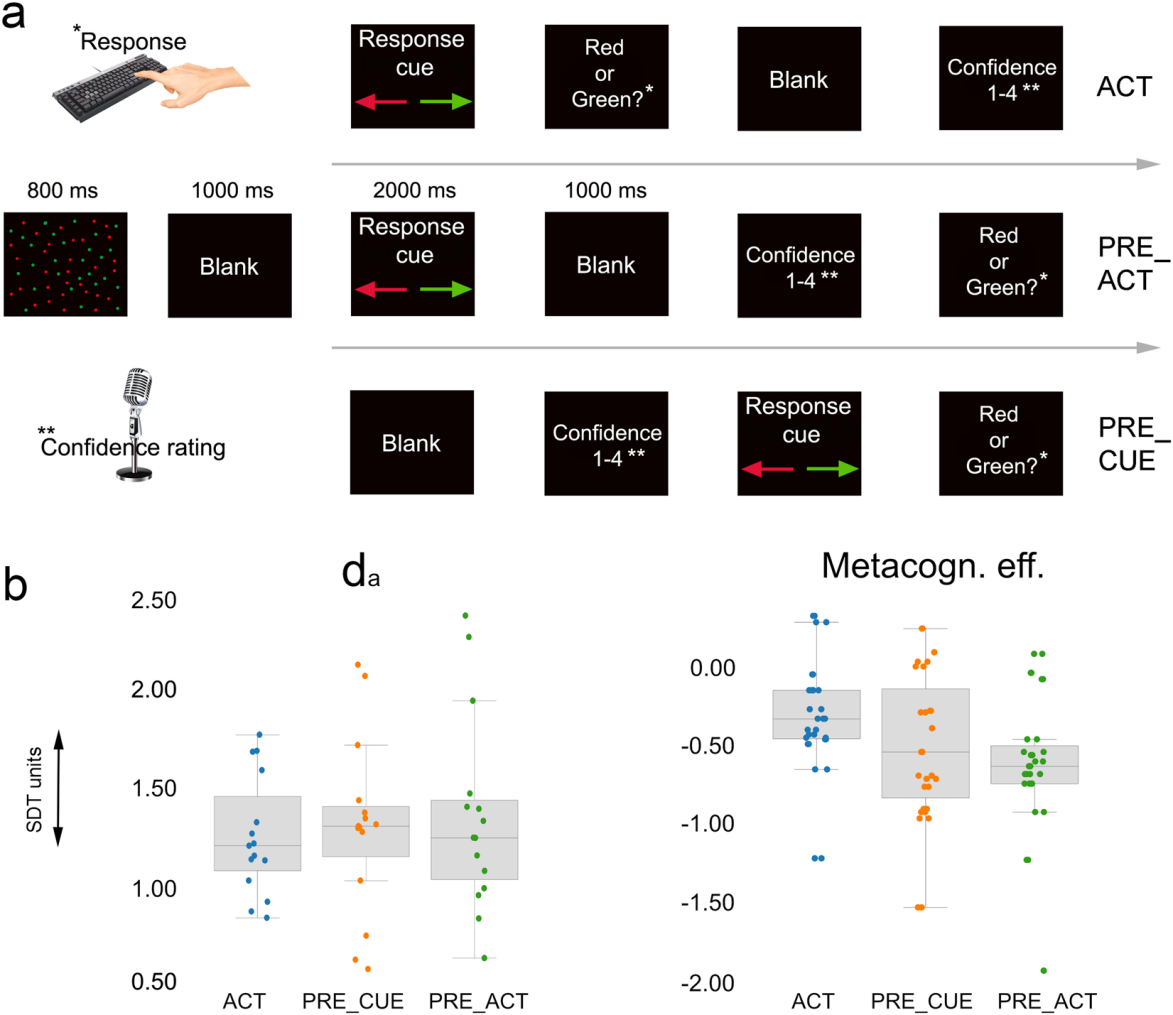
(**a**) Task design experiment 1. Participants had to decide whether the majority of randomly moving dots were red or green by pressing a left or right key. The key that mapped onto a ‘red’ or ‘green’ answer was signaled by a response cue on each trial. Verbal confidence ratings were recorded either at the end of each trial (ACT), or directly preceding the first-order response (PRE_ACT), or directly following stimulus presentation (PRE_CUE). In this way, in each condition a different amount of first-order action information was available at the moment metacognitive decisions were provided. (**b**) Behavioral results. Participants’ metacognitive efficiency decreased when action information was not available, while first-order performance remained unaltered. Error bars represent between-subjects standard error of the mean.

To determine whether action processes (i.e., response competition, ‘ease’ of action preparation^29^) contributed to the quality of metacognitive judgments, we varied the amount of first-order action information present at the moment metacognitive decisions had to be provided (see Fig. 1a). We constructed three conditions that differed in the moment participants had to provide their metacognitive judgment (see methods). In the first condition, participants provided verbal metacognitive judgments after the response cue and after the first-order response (ACT condition). In the second condition, metacognitive judgments were provided before the first-order response but after the presentation of the response cue (PRE_ACT condition). In the third condition, participants provided metacognitive judgments before presentation of the response cue and execution of the first-order response (PRE_CUE condition). We performed three repeated measures ANOVAs (the three conditions as levels) on first-order task performance (d_a_), metacognitive sensitivity (meta d_a_) and metacognitive efficiency (meta d_a_ - d_a_), respectively (see methods). Metacognitive sensitivity quantifies (in units of d_a_) how well a participant can discriminate correct from incorrect decisions on a first-order task. Metacognitive efficiency is the ability to discriminate between correct and incorrect decisions relative to different levels of first-order task performance. Because of the known influence of first-order task performance on metacognitive performance (meta d_a_), metacognitive efficiency is a measure of metacognitive performance that is more independent from variability in first-order performance^30^.

We found a significant effect of condition, specifically for metacognitive efficiency (*F*_*(2, 28)*_ = 4.04 p = 0.0029, η^2^ = 0.224). For both d_a_ (*F*_*(2, 28)*_ = 0.631 p = 0.540, η^2^ = 0.043) and meta d_a_ (*F*_*(2, 28)*_ = 1.882 p = 0.171, η^2^ = 0.118) no significant effects were observed. Next, we performed (one-tailed) t-tests to find out whether metacognitive efficiency decreased when response information was reduced. Results demonstrate that ACT and PRE_CUE significantly differed from each other *(t*_*(14)*_ = 2.45, p = 0.014, *d* = 0.663, BF_+0_ = 4.75), while no significant differences were observed between ACT and PRE_ACT (*t*_*(14)*_ = 1.65, p = 0.061, *d* = 0.426, BF_+0_ = 1.47) and PRE_ACT and PRE_CUE (*t*_*(14)*_ = 1.45, p = 0.085, *d* = 0.374, BF_+0_ = 1.13), see Fig. 1b. These findings suggest that participants’ capacity to distinguish accurate from inaccurate decisions improved when first-order response information was fully available (the ACT condition) compared to when such information was entirely unavailable (the actual response and response preparation). We did not observe differences in the average confidence level between the conditions (all ts < 0.753, ps > 0.464).

We aimed to prevent the influence of prolonged evidence accumulation introduced by differences in time between stimulus offset and response as much as possible by introducing a blank of 1 second after stimulus presentation in all three conditions^31^. However, it could still be possible that a longer time window to reflect on the perceptual decision could nonetheless influence performance independently of action information. In such a scenario of prolonged evidence accumulation we would expect the d_a_ to be higher in the ACT condition compared to the PRE_CUE and PRE_ACT conditions since evidence had more time to accumulate. To assess whether our experimental design was successful in preventing effects due to prolonged evidence accumulation, we post-hoc tested differences between d_a_ scores in ACT and PRE_ACT, and in ACT and PRE_CUE respectively (see Fig. 1b). We did not observe any significant d_a_ differences (ACT vs. PRE_ACT: *t*_*(14)*_=0.56, p = 0.584, BF_10_ = 0.301; ACT vs. PRE_CUE: *t*_*(14)*_=1.00, p = 0.334, BF_10_ = 0.403). These findings indicate that the presented blank after stimulus offset most likely eliminated effects of prolonged evidence accumulation.

### EEG results

**Figure 2:**
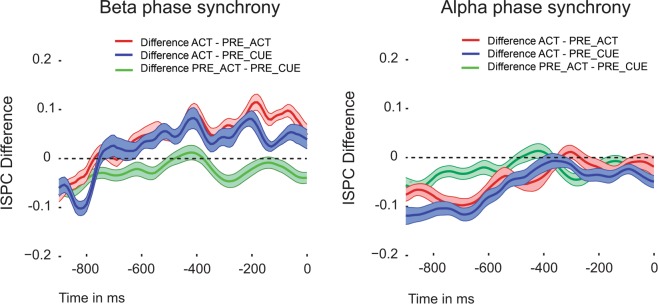
Functional connectivity. Functional connectivity (beta phase synchrony) between motor cortex and prefrontal cortex was higher in ACT where response information was available during metacognitive decision-making compared to PRE_ACT and PRE_CUE. No effects were observed for alpha phase synchrony. Shaded areas represent within-subjects standard error of the mean. Time zero refers to the onset of the metacognitive question (see Fig. 1).

In order to examine the neural mechanisms that support communication between motor areas and prefrontal regions during metacognitive decision-making, we assessed differences in interregional functional connectivity (beta phase synchrony) between the central frontal electrode Fz^9^(see methods) and motor channels C3 or C4 (depending on the hand that responded) in the 500 ms time window preceding participants’ metacognitive judgment. There was a significant effect of condition for changes in beta phase synchrony (Greenhouse-Geisser corrected: *F*_(1.29,18.19)_ = 8.434, *p* = 0.006, η^2^ = 0.376). Because oscillatory activity in the alpha band has also been closely linked to action mechanisms^32^, we explored whether differences between conditions in alpha phase synchrony could be observed. No effects were found for changes in alpha phase synchrony between conditions (*F*_(2,28)_ = 1.483, *p* = 0.244, η^2^ = 0.096); see Fig. 2. We found higher functional connectivity (beta phase synchrony) in ACT compared to PRE_ACT (*t*_*(14)*_ = 3.89, p = 0.002, *d* = 1.004, BF_10_ = 25.437) and PRE_CUE (*t*_*(14)*_ = 2.446, p = 0.028, *d* = 0.632, BF_10_ = 2.405). No differences were observed between PRE_ACT and PRE_CUE (*t*_*(14)*_ = 1.20, p = 0.250, *d* = 0.310, BF_10_ = 0.482).

**Figure 3:**
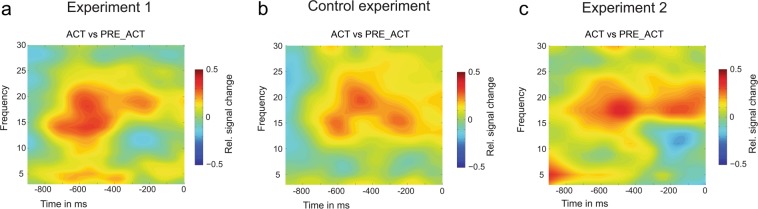
Time frequency results of experiment 1 (**a**), control experiment (**b**) and experiment 2 (**c**). In contrast to the functional connectivity results, we observed a similar pattern of enhanced beta power in all three experiments (including the control experiment), indicating that these beta power effects are unspecific to metacognitive decision-making. Time zero refers to the onset of the metacognitive question (see Fig. 1).

Next, we investigated whether functional connectivity changes (beta phase synchrony) were accompanied by changes in beta power in the central frontal channel Fz. Beta power was higher in ACT compared to PRE_ACT (*t*_*(14)*_ = 2.765, p = 0.015, *d* = 0.714, BF_10_ = 3.957), while no differences were found between ACT and PRE_CUE (*t*_*(14)*_ = 1.364, p = 0.194, *d* = 0.352, BF_10_ = 0.011); see Fig. 3a.

### Control experiment

**Figure 4:**
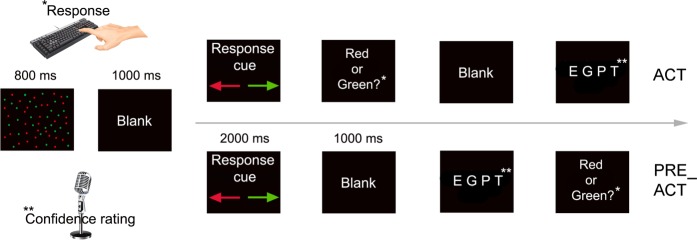
Task design control experiment. In the control experiment we replaced the metacognitive decision with a verbal response of a letter, while keeping the rest of the design identical to ACT and PRE_ACT of the first experiment.

**Figure 5:**
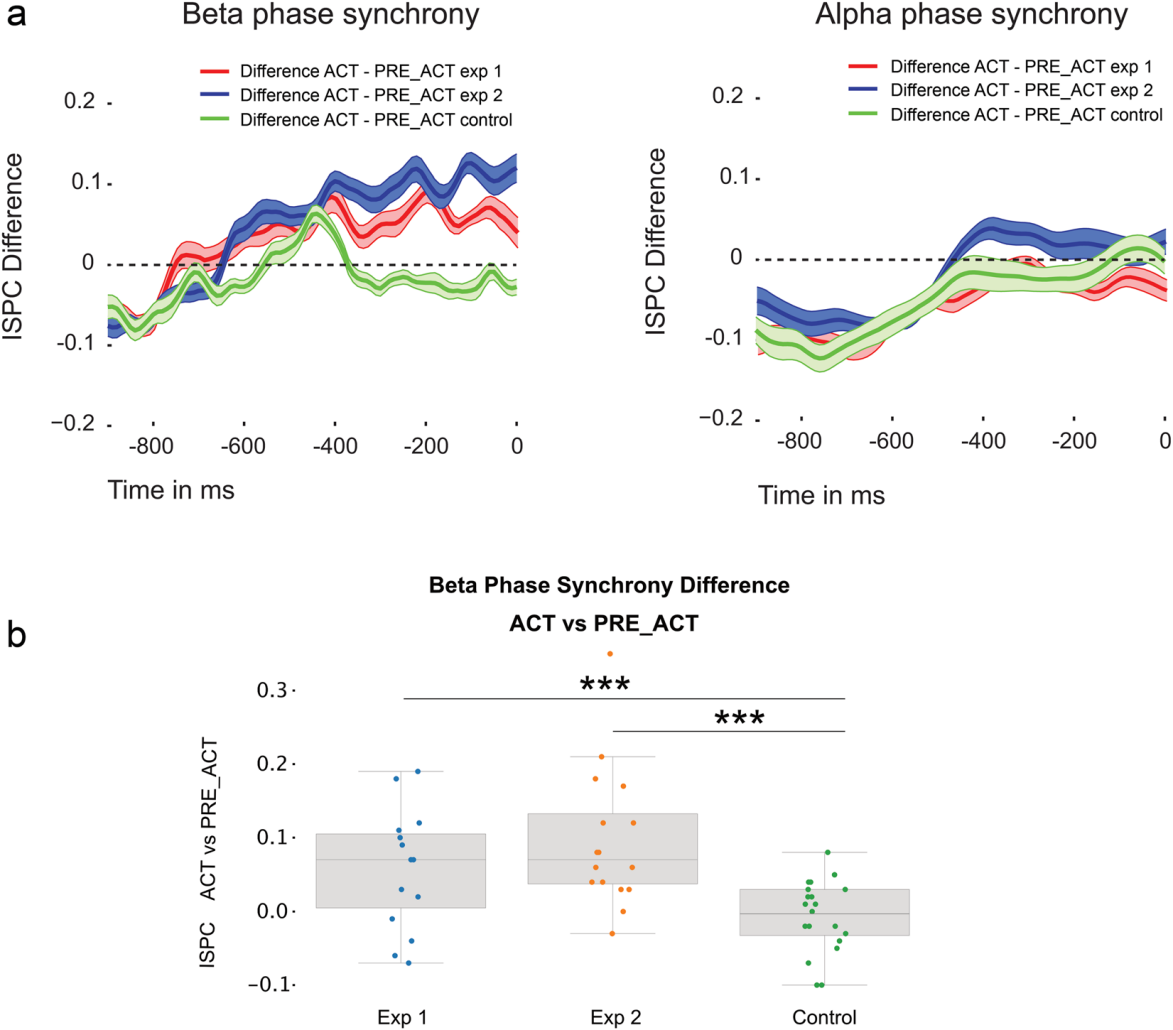
(**a**) Functional connectivity differences of beta (left) and alpha (right) phase synchrony. Similar to Fig. 2, we observed enhanced functional connectivity (beta phase synchrony) between motor cortex and central frontal cortex in ACT where response information was available during metacognitive decision-making compared to PRE_ACT. This effect was not observed in the control experiment where participants were not engaged in a metacognitive task. In all three experiments, no alpha phase synchrony differences were observed. Shaded areas represent within-subjects standard error of the mean. (**b**) Direct comparisons of the observed beta phase synchrony differences in all three experiments show that the effect is specific to settings in which metacognitive decisions are required. Error bars represent between-subjects standard error of the mean. Time zero refers to the onset of the metacognitive question (see Fig. 1).

In our EEG analyses, we attempted to minimize the effect of the mere presence of a motor response (the act of moving your finger) by focusing on the last 500 ms preceding the metacognitive judgment (see Fig. 1a). Nonetheless, EEG results observed in the first experiment could still be influenced by epiphenomenal/lingering motor activity caused by pressing a button in ACT versus not having pressed a button in PRE_ACT and PRE_CUE. We thus repeated the first experiment (ACT and PRE_ACT) while replacing the verbal confidence judgment with a verbal report of a random letter (see Fig. 4). In this way, we were able to find out whether the observed beta effects (phase synchrony/power) were related to epiphenomenal motor activity or whether this was instead specifically linked to metacognitive judgments. In the control experiment, no differences in first-order performance (d_a_) between the two conditions were observed (*t*_*(18)*_ = 0.164, p = 0.872, *d* = 0.038, BF_10_ = 0.240; Mean d_a_ condition 1 = 0.99, SD = 0.45; Mean d_a_ condition 2 = 0.97, SD = 0.44). In contrast to the first experiment, we did not observe a significant difference in functional connectivity between ACT and PRE_ACT (beta phase synchrony*: t*_*(18)*_ = 0.475, p = 0.641, *d* = 0.109, BF_10_ = 0.263; alpha phase synchrony: *t*_*(18)*_ = 0.511, p = 0.615, *d* = 0.117, BF_10_ = 0.267), see Fig. 5a. Similarly to the first experiment, however, we did observe a difference in beta power between ACT and PRE_ACT (*t*_*(18)*_ = 5.098, p < 0.001, *d* = 1.201, BF_10_ = 311.7), see Fig. 3b. These findings indicate that the increase in functional connectivity (beta phase synchrony) between frontal and motor areas is not merely caused by epiphenomenal first-order response activity, but seems instead to be connected to the metacognitive processes that follow first-order responses. In contrast, beta power differences between the conditions in the current experiments seem to be non-specific to what happens after the first-order response: we observed beta power differences when a metacognitive judgment had to be provided as well as when a random letter had to be reported.

### Experiment 2

**Figure 6:**
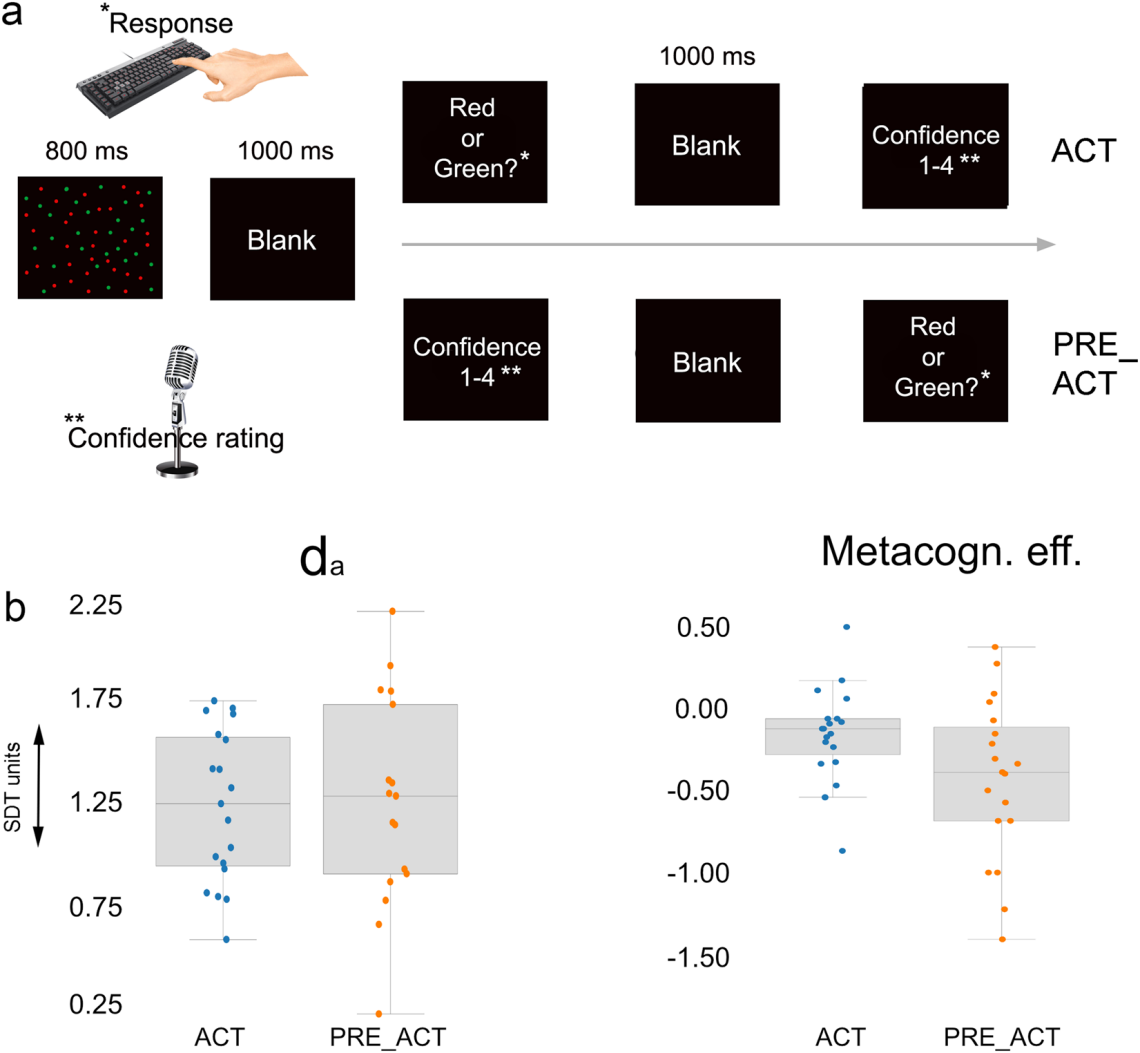
(**a**) Task design experiment 2. In the second experiment we omitted the response cue, while keeping the rest of the design similar to experiment 1. (**b**) Behavioral results. We replicated our findings from the first experiment and observed that metacognitive efficiency decreased when action information was absent, while first order performance remained unaffected. Error bars represent between-subjects standard error of the mean.

To find out if we could replicate the findings from the first experiment and to investigate whether the strength of the stimulus-response mapping influenced the strength of the observed behavioral and EEG effects, we recorded behavioral data and EEG signals during a second experiment in which we omitted the response cue (see Fig. 6a). As such, the experiment was similar to the first experiment with the exceptions that the stimulus-response mapping was kept stable across the entire experiment, and that the PRE_CUE condition was no longer present.

### Behavior

We performed (one-tailed) t-tests to find out whether metacognitive efficiency decreased when action information was absent. We replicated findings from the first experiment (though the statistical effect is small) and found increased metacognitive efficiency when response information was available (ACT) compared to PRE_ACT in which this information was absent *(t*_*(18)*_ = 2.134, p = 0.023, *d* = 0.490, BF_+0_ = 2.89). No significant differences were observed between conditions for d_a_ scores (*t*_*(18)*_ = 0.713, p = 0.758, *d* = 0.164, BF_+0_ = 0.151) or meta d_a_ scores (*t*_*(18)*_ = 1.622, p = 0.061, *d* = 0.372, BF_+0_ = 1.337), see Fig. 6b. In this experiment, we did observe a consistent lower level of confidence in ACT (mean = 2.63, SD = 0.433) compared to PRE_ACT (mean = 2.70, SD = 0.431), *t*_*(18)*_ = 2.999, p = 0.012, *d* = 0.642, BF_10_ = 4.17.

Although our design is not well suited to investigate differences in reaction times (due to the 1 s blank that preceded each first-order response, see Fig. 6a), we nonetheless tested whether RT differences existed between ACT (Mean = 491 ms, SD = 61) and PRE_ACT (Mean = 502 ms, SD = 70) that could accompany the observed difference in confidence level between the conditions. We found no differences in RT between both conditions (*t*_*(18)*_ = 1.970, p = 0.064, *d* = 0.452, BF_10_ = 1.163).

### EEG results

In the second experiment we repeated the analyses from the first experiment by focusing on functional connectivity differences between ACT and PRE_ACT. We replicated our previous findings and observed higher functional connectivity (beta phase synchrony) in ACT compared to PRE_ACT (*t*_*(15)*_ = 4.038, p = 0.001, *d* = 1.009, BF_10_ = 36.003; alpha phase synchrony: *t*_*(15)*_ = 0.881, p = 0.392, *d* = 0.22, BF_10_ = 0.358), see Fig. 5a. We also observed higher beta power in ACT compared to PRE_ACT (*t*_*(15)*_ = 2.639, p = 0.019, *d* = 0.660, BF_10_ = 3.269, see Fig. 3c), however, due to a similar beta power effect observed in the control experiment, it is highly unlikely that the beta power effects are the result of our experimental manipulation.

### General results

**Figure 7:**
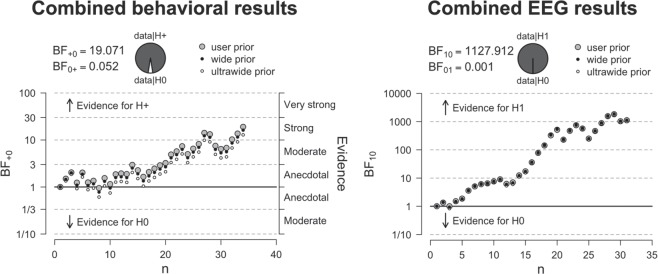
Combined results. When combining the data from experiment 1 and 2 we find strong evidence for increased metacognitive efficiency when action information is available during metacognitive decision-making. Similarly, strong evidence is observed for increased functional connectivity (beta phase synchrony) between motor channels and central frontal regions when action information is available during metacognitive decision-making.

In order to determine the overall effect of action processes on metacognitive efficiency, we grouped the data from the first and second experiment together (see methods) using Bayesian statistics, which make it possible to meaningfully aggregate subjects and/or experiments in a post-hoc manner. We therefore grouped PRE_ACT and PRE_CUE from experiment 1 so as to create two conditions, as in experiment 2. We observed strong evidence for higher metacognitive efficiency (BF_+0_ = 19.151, see Fig. 7) when action information was available during metacognitive judgments. Note that the combined effect is much stronger than the weak behavioral effects observed in each individual study, suggesting the need for large enough sample size. Future studies investigating changes in metacognitive performance could benefit from such a larger sample size, and from using a longer lasting staircase procedure for second-order performance as well as first-order performance, preventing the exclusion of participants.

**Figure 8:**
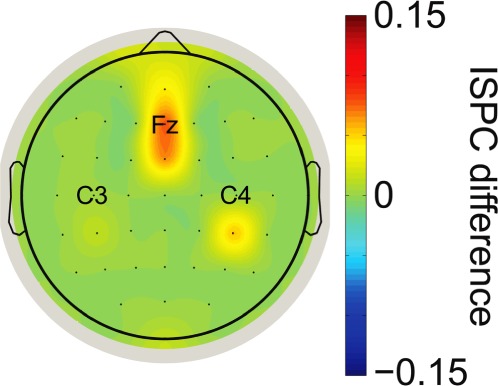
Topoplot of the combined functional connectivity effect (ACT vs. PRE_ACT). For illustration purposes we plotted beta phase synchrony differences between ‘seed’ electrode C3/C4 and other electrodes to show the spatial distribution of the observed effect.

To test whether functional connectivity differences between ACT and PRE_ACT differed between the experimental and control experiment, we directly compared ACT and PRE_ACT differences with each other^33^ using independent sampled t-tests. In all experiments we subtracted values from PRE_ACT from ACT. Again we averaged PRE_ACT and PRE_CUE from experiment 1 and subtracted that from the ACT condition. We observed significantly greater differences in the experimental conditions compared to the control condition (first experiment vs. control experiment: *t*_*(32)*_ = 2.904, p = 0.007, *d* = 1.003, BF_10_ = 6.901; second experiment vs. control experiment: *t*_*(33)*_ = 4.057, p < 0.001, *d* = 1.377, BF_10_ = 87.51), see Fig. 5b. When examining the combined data from the first and second experiment with respect to functional connectivity, we find strong evidence for greater beta phase synchrony between motor and central frontal regions when action information is available at the moment of metacognitive decision-making (BF_+0_ = 1127.912, see Fig. 7 & 8).

## Discussion

Decision-making is typically accompanied by an estimate about the quality of one’s choices, actions or performance. Adequate metacognition is not only important in everyday life settings (e.g., whether you can assess whether you are still able to drive safely on a long trip, or knowing what you know while studying for an exam), but can even be critical in certain situations (e.g., in case of medical decisions, or decisions made by a flight controllers). Despite its importance, it remains unclear how metacognition emerges, and what kind of information is used to determine the quality of our decisions.

Here, we investigated whether first-order action information could inform second-order (metacognitive) decisions. Specifically, we studied whether reducing available first-order response information at the moment second-order decisions had to be provided affected metacognitive performance in a color discrimination task. Further, we investigated whether functional connectivity between motor regions and prefrontal cortex could be a candidate to convey action information during metacognitive decision-making. Results demonstrate that metacognitive efficiency slightly decreased when first-order action information was reduced at the moment metacognitive decisions had to be provided. We replicated our findings in a second experiment and showed that the effect was small but robust to changes in the experimental design (see Figs. 1b, 6b & 7). Similarly, we found converging electrophysiological evidence that functional connectivity between motor areas and prefrontal cortex increases during metacognitive decision-making when action information is available (see Fig. 2 & 5). In a control experiment, we demonstrated that this effect was not related to lingering response activity, but in fact specific to metacognitive processes following first-order decisions (Fig. 5). Combined analyses of the three experiments provide converging evidence for the contribution of action information in metacognitive decision-making.

### Models of metacognitive decision-making

In lab settings, metacognition is typically studied by asking participants to make a decision about a stimulus (e.g., the motion direction of a cloud of moving dots, the orientation of a grating), after which they are asked to provide the level of confidence in their decision being correct. Previously, it has been shown that manipulating stimulus parameters (evidence strength and evidence reliability) affects confidence judgments^34^ during perceptual decision-making, suggesting similar (sensory) evidence processing mechanisms support first- and second-order decision-making. Similarly, in signal-detection-like models, the distance of the decision variable from a criterion represents a level of confidence^4,6,35,36^. The time between the decision and presentation of sensory evidence could in such cases result in discrepancies between first- and second-order decisions, due to prolonged accumulation of evidence^10,37,38^. Alternatively, different sources or quality of information could contribute to first- and second-order decisions^39,40^, resulting in different first- and second-order performance^12^. With respect to the latter, we previously demonstrated that sensory evidence contributing to first-order decision-making does not similarly support metacognitive decision-making. Variance in first-order performance was driven by different stimulus features compared to variance in metacognitive performance. These findings indicated that sensory evidence used for first-order performance differed from information used for metacognitive judgments^9^. Maniscalco and Lau recently compared models describing discrepancies between first- and second-order decisions during a visual masking task. They compared models which depict first- and second-order decision-making as supported by similar sources of information (single channels models) with dual channel models, which describe two processing streams giving rise to first- and second-order task performance; and hierarchical models, which presume that a late processing stage monitors the state of sensory processing. Their results demonstrated that dissociations between first- and second-order performance are best captured by hierarchical models. Hierarchical models of metacognition propose that sensory evidence used for first-order performance can become susceptible to accrual of noise and signal decay over time and due to further processing^12^. As such, the experimental design itself can be important as the first-order response is typically given closer in time to stimulus offset compared to the second-order response. Over time, various factors can contribute to a loss in strength of the sensory signal. For instance, further neural processing of the sensory signal could result in the accumulation of noise when arriving at the stage at which this information is being used by the metacognitive system^12,39^. Therefore, our design not only manipulated the amount of available “action information” but additionally also manipulated the (potential) level of accumulated noise/signal decay. However, in our design the effect of signal decay and noise should counter any beneficial effect of action information available at a later processing stage: On the one hand additional information becomes available for the metacognitive system at a later processing stage, but on the other hand the sensory evidence has most likely become degraded^9,12^. In the current experiments, we observed slight improvements of metacognitive efficiency when the metacognitive judgment was made with more time in between stimulus offset and the second-order response. In order to tease these different factors apart it would be interesting to combine our previously used experimental design^9^ with an adaptation of the current design in order to investigate signal decay/noise accumulation in combination with the contribution of action information.

Another factor that has to be taken into account reflects observations indicating that the level of confidence is mainly driven by response-congruent evidence, and appears to be less sensitive to response-incongruent evidence^41,42^. From such a perspective, a confidence judgment made prior to the first-order decision could be based on the strength of evidence of each response alternative, whereas a confidence judgment made after to the first-order decision would be dominated by response-congruent evidence. In our task, we instructed our participants to provide a level of confidence of the to-be-made decision, thereby stimulating a commitment to one decision alternative prior to the second-order decision. However, we did not assess the exact moment of commitment to the perceptual decision directly, leaving it an open empirical question how information from different response alternatives contributes to confidence judgments when shifting the order within a trial.

Fleming and Daw^19^ recently put forward a framework in which confidence operates as a second-order computation about one’s own performance. While first-order models are able to reproduce the above-described relationship of confidence and stimulus parameters, their second-order model accommodates the present findings that action information influences metacognitive performance and metacognitive bias. The second-order framework predicts that action affects confidence ratings, in the sense that it decreases overall confidence and enhances metacognitive performance. In the current experiments we observed this pattern in our behavioral results. In two experiments, we demonstrated that metacognitive efficiency increased when first-order action information became available for second-order decision-making. In addition, we observed a (somewhat counterintuitive) decrease in confidence when metacognitive judgments followed first-order responses in the second experiment, as predicted by the second-order model^19^. We did not observe differences in overall confidence in the first experiment. It could be that trial-by-trial alternations of stimulus-response mappings in the first experiment tampered the effect on metacognitive bias shifts. Previously, it was found that participants’ metacognitive bias shifted when they learned motor sequences in a blocked design compared to when sequences were interleaved^43^. These findings suggest that the current ease of stimulus-response mappings affected metacognitive bias. In that sense, it would be interesting for future experiments to assess whether/how manipulation of ease or the integrity of first-order responses influences metacognitive behavior.

### Beta oscillations

Beta oscillations are classically linked to sensory and motor processing^20,28^. During preparation and execution of movements, beta band activity typically decreases initially, followed by an increase in beta power^44^. For instance, an upcoming action could be reliably predicted several seconds prior to response execution, based on lateralization of beta band activity in motor regions, linking beta band activity to the unfolding of an action^21^. It has been suggested that beta activity reflects the maintenance of an existing motor set whilst weakening processing of new actions^45^. Interestingly, beta synchronization has been associated with the correctness of an action and has been shown to follow motor errors or after observing the motor errors of others^46,47^. Recently, the importance of beta oscillations has been demonstrated beyond the sensorimotor domain, extending to visual perception^27,48,49^, working memory^50^, long-term memory^51^, and decision-making^21,24,52^. It has been proposed that beta oscillations support long-range neuronal interactions^25,53,54^, thereby maintaining a current cognitive set, sensorimotor state or the so-called ‘status quo’^26^. In this way, the up or down regulation of beta depends on whether the ‘status quo’ is prioritized over novel incoming signals. Recently, Spitzer and Haegens^28^ extended the role of beta oscillations further, advocating a role of beta in the awakening of a (endogenous) cognitive set, depending on current task demands.

In the current study, we found increased phase synchrony in the beta band between motor channels and central frontal regions (electrode Fz) specifically when a metacognitive decision followed the first-order response. Critically, when task demands changed and a metacognitive judgment was not required, beta phase synchrony differences between conditions disappeared. In line with the above-proposed role of beta oscillations, our beta phase synchrony findings indicate that task demands (the metacognitive task) resulted in the maintenance of first-order action information (e.g., response fluency, response competition strength). It would be interesting to investigate what role explicitly asking for a metacognitive judgment has on beta band activity. If we assume that decisions are naturally accompanied by an estimate about the quality of an action or choice, it could be that by explicitly asking for such an estimate after a short time interval we could have prolonged or boosted a naturally occurring more transient event (for a similar discussion in consciousness research^55^). Indeed, beta phase synchrony effects in the control condition initially seem to mimic those observed in the other two experiments, only starting to deflect in the period preceding the metacognitive judgment. It would be interesting to test ‘naturally occurring’ metacognitive processes in future experiments, thereby using observed neural markers of explicitly probed metacognitive processes^9,56–58^^,^.

### Motor activity and metacognition

The present results indicate a contribution of first-order motor response information in metacognitive decision-making. Previously, Wenke and colleagues^29^ demonstrated that participants were sensitive to conflicting motor activity (response competition) induced by subliminal information. In their study the “ease” or “smoothness” of action selection in a visual reaction-time task was manipulated by presenting a subliminal response prime that was congruent to one out of two action possibilities. Results demonstrated that action priming influenced the sense of control over action consequences following the response. Other work indicates that metacognitive experience of response competition is crucial for triggering cognitive adaptation^59,60^. Further, it has been shown in the memory domain that the experience of motor fluency is used as a cue that affects metamemory^61,62^.

Recently, it has been shown that perceptual decisions were biased by the amount of motor effort it took for participants to make the response^62^. In this study, participants’ decision was biased towards the least effortful motor response. These findings demonstrate that the ease to act on a decision might influence the decision itself. However, it seems that metacognitive awareness of effort or of task demands is necessary for the development of such a decision bias^63^. In the current experiments, results indicate that participants could be sensitive to response competition, the fluency or ease of the first-order response^60,64^ when computing an estimate about the quality of the decision.

Alternatively, motor activity could provide insight into the mechanisms of the unfolding perceptual decision. Recent studies demonstrated that evidence accumulation processes ‘echo’ in activity in motor regions^21^. As such, perceptual and cognitive states could be reflected in the motor system^65,66^ and be used to inform metacognitive decisions.

### Prefrontal cortex and metacognition

Previous work demonstrated that lesions to prefrontal cortex affect metacognitive performance without altering first-order decision-making^67,68^. Similarly, disrupting prefrontal activity via theta burst stimulation has been shown to selectively alter metacognitive performance^69–71^ (but see^72,73^). The detection of erroneous behavior, a key aspect of metacognition^5,19^, has been strongly linked to a rapidly emerging central frontal negativity in the EEG signal (error-related negativity^74^), thought to reflect coordinated theta oscillatory mechanisms^75–79^. In addition, theta has been implicated in learning, feedback processing, and action monitoring^77,80–85^. Recently, fluctuations in prefrontal theta band activity has been linked to fluctuations in metacognitive performance^9,57,86^. Taken together, these findings suggest that frequent exposure to external feedback, learning from one’s correct and incorrect decisions induces a shift in which error detection, initially elicited by external feedback (or observing the consequences of our decisions), is shifting towards the use of internal simulations of stimulus-response contingencies. This internally processing of the probabilities of our actions towards outside events and their most likely outcomes^5,87–89^ could be used to adapt future behavior. In such a way, metacognition could be seen as an internalization of external feedback processing and error monitoring, employing similar neural mechanisms^57,90,91^.

**Figure 9:**
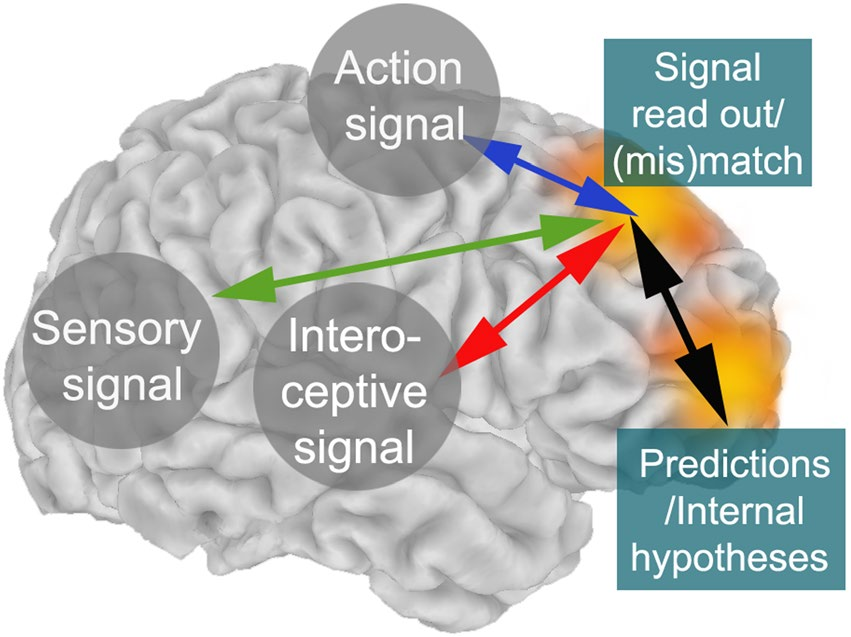
Sensory, interoceptive and action signals are read out in central frontal cortex. Anterior prefrontal cortex provides predictions about the “state of the world” and the “state of the decider” when a decision is made. Central frontal theta oscillations serve as a mechanism to broadcast the need for control in response to the estimate about the quality of the decision.

It has been previously proposed that next to perceptual evidence, inferences about “the state of the decider” (i.e., one’s own actions^19^, and prior or global estimates of performance^92,93^) are important for metacognitive decision-making. In addition, to adequately compute an estimate about the quality of a decision it is necessary to know the broader task context or infer “the state of the world” (i.e., value for an action at a certain state of the (task) environment) at the moment of the decision^94–97^. Recently, the orbitofrontal cortex has been linked to inferring such “states of the world” during decision-making^95,98^. As such, central frontal regions and anterior frontal areas could play distinct roles in metacognitive decision-making^71^. Figure 9 illustrates how sensory, action and interoceptive signals could be integrated in central frontal regions, interacting with anterior prefrontal regions providing inferences about the state of the world^94,95^ and the state of the decider^19,93^ when computing an estimate about the quality of a decision.

### Limitations

In the current study, we focused on functional connectivity changes between motor and prefrontal regions. However, the current neural measurements (EEG) lack spatial specificity to make strong claims about neural sources. It would therefore be necessary to replicate our findings using alternative methods (e.g. fMRI) that have a higher spatial resolution.

We used a staircase performance prior to the experimental blocks to determine appropriate task settings. Despite our efforts we had to exclude participants based on first- and second-order task performance. In future studies it might be useful to use a longer staircase period to eliminate learning effects, and employ a staircase procedure for second-order task performance in addition to first-order performance.

## Conclusion

Monitoring and evaluation of one’s own performance is crucial for adept behavior. However, how metacognition emerges is still hotly debated^10,12,19^. In a series of three experiments, we demonstrated that manipulations of available action information affected metacognitive performance. Concurrent EEG recordings showed that functional connectivity between prefrontal regions and motor areas increased after a first-order response, specifically when a metacognitive judgment was required. Together with previous findings^9,17,67^, our results paint a picture of metacognition as a second-order process that integrates sensory and action information.

## Materials and Methods

### Participants

Twenty-five participants (15 females, mean age = 21.1, SD = 4.82) took part in experiment 1, twenty-nine participants (18 females, mean age = 22.1, SD = 2.65) in experiment 2, and twenty (13 females, mean age = 21.6, SD = 3.87) in the control experiment. Participants received financial compensation for their participation in this experiment. All participants had normal or corrected-to-normal vision and were naïve to the purpose of the experiment. All procedures complied with international laws and institutional guidelines and were approved by the local Ethics Committee of the Université Libre de Bruxelles, department of Psychology. All participants provided their written informed consent prior to the experiment.

### Task design

A field of 600 green and red moving dots was centrally presented (250*250 pixels) on a Dell 17 monitor with a refresh rate of 60 Hz. The monitor was placed at a distance of ~57 cm in front of each participant so that the collection of moving dots subtended a visual angle of 6.6°. Crucially, on each trial a majority of the 600 dots (on average 315.11 dots, SD = 6.76) was either green or red. Participants were instructed to determine what color (red or green) was predominant on each trial by pressing a left (~) or right (/) key. The level of difficulty was determined for each participant individually by using a one-up- two-down staircase procedure in steps of 0.5% of total number of dots before the start of the experiment. After two consecutive correct responses, the difference between the total number of red vs. green dots was reduced by 0.5% (3 dots). During the staircase procedure, each participant performed a total of three blocks (one block of each condition in experiment 1, and one block of each condition in the control and second experiment plus a block randomly picked between condition 1 and 2) in order to assess the level of difficulty that resulted in a stable level of performance set at 71% correct. The stimulus was presented for 800 ms, and at any moment during stimulus presentation a total of 600 dots were displayed. Each trial started with a blank screen (jittered between 1000–1500 ms, in steps of 100 ms) on which a fixation cross was centrally presented. After stimulus presentation a blank was presented for 1000 ms to avoid the influence on prolonged evidence accumulation^5,31^.

### Experiment 1

In the first experiment, we created three conditions by varying the amount of available action information at the moment a metacognitive decision had to be provided (Fig. 1a). In condition 1, the stimulus and blank screen were followed by a response cue (2000 ms), instructing participants whether the left or right button corresponded to the answer “green” or “red” (Fig. 1a). The stimulus-response mapping was randomized so that in approximately half of all trials the left response button signaled ‘red’ and in approximately the other half of the trials it signaled ‘green’. This randomized stimulus-response mapping prevented participants from preparing their response immediately after the visual stimulus had appeared and enabled us to disentangle motor preparation from motor action in both our behavioral and EEG analyses. After the presentation of the response cue, participants were asked to indicate whether the majority of the dots were green or red by pressing the corresponding button with their left or right index finger. Next, participants had to provide a metacognitive judgment about their decision by indicating their level of confidence in being correct on a labeled scale from 1–4, where 1 indicated being very uncertain and 4 being very certain that their first-order response was correct. Participants were encouraged to use the whole range of the scale. Participants verbally reported their confidence rating in order to link the manual motor response exclusively to the first-order decision (red-green decision). A microphone registered all verbal responses using speech recognition software in Presentation (Neurobehavioral Systems, version 18.1), allowing automatic recording of verbal responses. To ensure an accurate transcription of the responses, we set a threshold level of certainty (0.8). Flagged trials below 0.8 certainty were checked manually and corrected if necessary (4% of all trials).

Critically, confidence ratings were given at different points in the trial sequence depending on the condition. Typically, confidence ratings are given after the first-order task response (ACT). However, in this experiment we manipulated the amount of action information (i.e., response execution, action preparation) available for metacognitive decisions by varying the position of metacognitive judgments in a trial. In PRE_ACT, metacognitive judgments had to be provided before the first-order response (after the response cue), while in PRE_CUE metacognitive decisions had to be made prior to the first-order response and presentation of the response cue. This resulted in two conditions in which action information was minimal (response preparation) or absent at the moment the second-order (metacognitive) decision was made.

### Control experiment

In the control experiment, we investigated whether observed EEG results were specific to metacognitive processes, by studying the non-specific effect of epiphenomenal/lingering motor activity from first-order responses. Therefore, we used a similar task design as used in the first experiment. Critically, in the control experiment participants were instructed to verbally report one randomly chosen letter out of four presented letters (‘E, ‘G’, ‘P’, ‘T’), instead of providing a confidence rating. Here, we focused on differences between ACT and PRE_ACT, since we did not observe behavioral and functional connectivity differences between PRE_ACT and PRE_CUE in the first experiment, see Fig. 1b.

### Experiment 2

In the second experiment, the response cue was removed in order to establish reliable stimulus-response mappings throughout the experiment. The rest of the design was kept similar to that of the first experiment (Fig. 1c).

### Behavioral analyses

In the present experiment, we aimed to investigate whether we could observe changes in metacognitive (second-order) performance depending on experimental condition. We therefore used a staircase procedure before starting the experiment (see above) and employed an exclusion criterion of d_a_ or meta d_a_ > 0.5 and <2.0 observed in the ACT condition (metacognitive performance is typically measured after first-order responses) in order to avoid floor and ceiling effects. As stated above, the aim of this study was to investigate fluctuations in metacognitive performance and such floor and ceiling effects would preclude the aim of the experimental design. Additionally, by filtering the data we tried to avoid potential issues with respect to the structure of the data (.e., by having little correct/incorrect trials in the data that are necessary for meta-d’ measures; for a recent discussion see^72^). To illustrate, nine participants that were excluded from the last experiment had a mean meta d_a_ of −0.02 in the ACT condition. This means that those participants performed the second-order task at chance level. One possible explanation could be that the way we recorded the second-order response (an English verbal report) was challenging for some of the excluded (native French-speaking) participants, thereby avoiding more difficult pronounceable answers.

For analyses, 15 participants were included in the first experiment, 18 in the control experiment and 19 in the second experiment. In order to find out whether first-order and metacognitive performance differed we calculated first-order task sensitivity (because the data was split into three conditions we calculated d_a_^35^, metacognitive sensitivity (meta-d_a_) and metacognitive efficiency (meta d_a_ – da^30,32^), for each condition separately. First-order task sensitivity (d_a_) and metacognitive sensitivity (meta-d_a_) are bias-free measures of the ability to distinguish two signals from each other and the ability to distinguish between correct and incorrect decisions, respectively (both in units of first-order d_a_). Metacognitive efficiency reflects metacognitive sensitivity relative to different levels of first-order task performance, which is important because metacognitive sensitivity is known to be influenced by first-order task performance^30^.

We performed three repeated measures analyses of variance (ANOVA) on first- and second-order task performance (d_a_, meta-d_a_, and metacognitive efficiency) with condition as the independent variable. All behavioral analyses were performed using JASP (Version 0.8.3.1), Matlab (Matlab 12.1, The MathWorks Inc.), type 2 SDT scripts^99^ and SPSS (IBM SPSS Statistics, 22.0). For the Bayesian analysis in JASP a Cauchy prior distribution centered around zero was used with an interquartile range of r = 0.707.

### EEG measurements and analyses

EEG was recorded and sampled at 1048 Hz using a Biosemi ActiveTwo 64-channel system, with four additional electrodes for horizontal and vertical eye-movements, each referenced to their counterpart (Biosemi – Amsterdam, The Netherlands). High-pass filtering (0.5 HZ), additional low-pass filtering (100 HZ) and a notch filter (50 HZ) were used. Next, we down-sampled to 512 Hz and corrected for eye movements on the basis of Independent Component Analysis^100^. The data was epoched −1.5 s to + 0.5 sec preceding confidence judgments. We removed trials containing irregularities due to EMG or other artifacts by visually inspecting all trials. To increase spatial specificity and to filter out deep sources we converted the data to spline Laplacian signals^100,101^. We used a sliding window Fourier transform^102^, window length: 400 ms, step size: 50 ms, to calculate the time-frequency representations of the EEG power (spectrograms) for each channel and each trial. We used a single Hanning taper for the frequency range 3–30 Hz (frequency resolution: 2.5 Hz, bin size: 1 Hz^27^). To examine the way information might be distributed during metacognitive decision-making, we assessed measures of interregional functional connectivity in the beta range. In our previous study, we specifically observed effects in prefrontal channel Fz related to metacognitive performance^9^. Therefore, we specifically examined consistencies of the difference of time–frequency phase values between motor channels (C3/C4, depending on the hand that responded) and central frontal electrode Fz (Intersite Phase Clustering (ISPC)^25,38^) in the 500 ms time period immediately preceding the confidence judgment (see Fig. 1). We used ISPC measurements to determine whether reducing the amount of motor information available at the moment of confidence judgments changed the level of functional connectivity (i.e., alpha/beta phase synchronisation) between central prefrontal^9^ and motor regions. In experiment 2 three participants had to be excluded from further EEG analyses due failed EEG recordings.

Power modulations were characterized as the percentage of power change at a given time and frequency bin relative to baseline power value for that frequency bin. The baseline was calculated as the mean power across the pre-stimulus interval (from −0.3 to 0 s relative to stimulus onset). All signal processing steps were performed using Brain Vision Analyzer (BrainProducts) and Matlab (Matlab 12.1, The MathWorks Inc.), **X** code^103^ and Fieldtrip^104^.

### Significance

Monitoring and control of our decision process is a critical part of every day decision-making. When feedback is not available, metacognitive skills enable us to modify current behavior and adapt prospective decision-making. Here, we investigated what kind information is being used to compute an estimate about the quality of our decisions. Results indicate that during perceptual decision-making, information about one’s actions towards perceptual events is being used to evaluate the quality of one’s decisions. EEG results indicate that functional connectivity between motor regions and prefrontal cortex could serve as a mechanism to convey action information during metacognitive decision-making. Considered together, our results demonstrate that post-decisional information contributes to metacognition, thereby evaluating not only what one perceives (e.g., strength of perceptual evidence) but also how one responds towards perceptual events.

## Data Availability

The datasets generated during and/or analyzed during the current study are available from the corresponding author on reasonable request. The scripts and toolboxes used for analyzing the data can be downloaded at: TF analyses: http://www.fieldtriptoolbox.org/, SDT: http://www.columbia.edu/~bsm2105/type2sdt/ Statistics: https://jasp-stats.org/

## References

[1] Morales J, Lau H, Fleming SM (2018). Domain-General and Domain-Specific Patterns of Activity Supporting Metacognition in Human Prefrontal Cortex. J. Neurosci..

[2] Vaccaro AG, Fleming SM (2018). Thinking about thinking: A coordinate-based meta-analysis of neuroimaging studies of metacognitive judgements. Brain Neurosci. Adv..

[3] Rouault, M., Mcwilliams, A., Allen, M. G. & Fleming, S. M. Human metacognition across domains: insights from individual differences and neuroimaging. *Pers. Neurosci*. 1–28 (2018). 10.1017/pen.2018.16 PMC6217996 30411087

[4] Kiani R, Shadlen MN (2009). Representation of Confidence Associated with a Decision by Neurons in the Parietal Cortex. Science (80-.)..

[5] Yeung N, Summerfield C (2012). Metacognition in human decision-making: confidence and error monitoring. Philos. Trans. R. Soc. B Biol. Sci..

[6] Fetsch CR, Kiani R, Newsome W, Shadlen MN (2014). Effects of Cortical Microstimulation on Confidence in a Perceptual Decision. Neuron.

[7] Wierzchoń M, Paulewicz B, Asanowicz D, Timmermans B, Cleeremans A (2014). Different subjective awareness measures demonstrate the influence of visual identification on perceptual awareness ratings. Conscious. Cogn..

[8] Fleming SM (2015). Action-Specific Disruption of Perceptual Confidence. Psychol. Sci..

[9] Wokke ME, Cleeremans A, Ridderinkhof KR (2017). Sure I’m Sure: Prefrontal Oscillations Support Metacognitive Monitoring of Decision Making. J. Neurosci..

[10] Berg RVD, Zylberberg A, Kiani R, Shadlen MN, Wolpert DM (2016). Confidence is the bridge between multi-stage decisions. Curr. Biol..

[11] Palser ER, Fotopoulou A, Kilner JM (2018). Altering movement parameters disrupts metacognitive accuracy. Conscious. Cogn..

[12] Maniscalco B, Lau H (2016). The signal processing architecture underlying subjective reports of sensory awareness. Neurosci. of Consci..

[13] Cisek P, Kalaska JF (2005). Neural correlates of reaching decisions in dorsal premotor cortex: Specification of multiple direction choices and final selection of action. Neuron.

[14] Maniscalco, B. *et al*. Tuned normalization in perceptual decision-making circuits can explain seemingly suboptimal confidence behavior. bioRxiv: 558858 (2019). 10.1371/journal.pcbi.1008779 PMC8032199 33780449

[15] Allen, M. *et al*. Unexpected arousal modulates the influence of sensory noise on confidence. *Elife* **5** (2016). 10.7554/eLife.18103 PMC5079750 27776633

[16] Urai AE, Braun A, Donner TH (2017). Pupil-linked arousal is driven by decision uncertainty and alters serial choice bias. Nat. Commun..

[17] Siedlecka M, Paulewicz B, Wierzchoń M (2016). But I Was So Sure! Metacognitive Judgments Are Less Accurate Given Prospectively than Retrospectively. Front. Psychol..

[18] Pasquali A, Timmermans B, Cleeremans A (2010). Know thyself: Metacognitive networks and measures of consciousness. Cognition.

[19] Fleming SM, Daw ND (2017). Self-evaluation of decision-making: A general Bayesian framework for metacognitive computation. Psychol. Rev..

[20] Pfurtscheller G, Lopes da Silva FH (1999). Event-related EEG/MEG synchronization and desynchronization: basic principles. Clin. Neurophysiol..

[21] Donner TH, Siegel M, Fries P, Engel AK (2009). Buildup of Choice-Predictive Activity in Human Motor Cortex during Perceptual Decision Making. Curr. Biol..

[22] Tzagarakis C, Ince NF, Leuthold AC, Pellizzer G (2010). Beta-Band Activity during Motor Planning Reflects Response Uncertainty. J. Neurosci..

[23] Donner TH (2007). Population Activity in the Human Dorsal Pathway Predicts the Accuracy of Visual Motion Detection. J. Neurophysiol..

[24] Haegens S, Nácher V, Luna R, Romo R, Jensen O (2011). α-Oscillations in the monkey sensorimotor network influence discrimination performance by rhythmical inhibition of neuronal spiking. Proc. Natl. Acad. Sci. USA.

[25] Siegel M, Donner TH, Engel AK (2012). Spectral fingerprints of large-scale neuronal interactions. Nat. Rev. Neurosci..

[26] Engel AK, Fries P (2010). Beta-band oscillations — signalling the status quo?. Curr. op. in neurobiol..

[27] Kloosterman NA (2015). Top-down modulation in human visual cortex predicts the stability of a perceptual illusion. J. Neurophysiol..

[28] Spitzer, B. & Haegens, S. Beyond the Status Quo: A Role for Beta Oscillations in Endogenous Content (Re)Activation. *eneuro* **4**, ENEURO.0170-17.2017 (2017). 10.1523/ENEURO.0170-17.2017 PMC5539431 28785729

[29] Wenke D, Fleming SM, Haggard P (2010). Subliminal priming of actions influences sense of control over effects of action. Cognition.

[30] Fleming, S. M. & Lau, H. How to measure metacognition. *Front. Hum. Neurosci*. **8** (2014). 10.3389/fnhum.2014.00443 PMC4097944 25076880

[31] Hebart, M. N., Schriever, Y., Donner, T. H. & Haynes, J.-D. The Relationship between Perceptual Decision Variables and Confidence in the Human Brain. *Cereb. Cortex* (2014). 10.1093/cercor/bhu181 25112281

[32] Brinkman L, Stolk A, Dijkerman HC, de Lange FP, Toni I (2014). Distinct roles for alpha- and beta-band oscillations during mental simulation of goal-directed actions. J. Neurosci..

[33] Nieuwenhuis S, Forstmann BU, Wagenmakers E-J (2011). Erroneous analyses of interactions in neuroscience: a problem of significance. Nat. Neurosci..

[34] Boldt A, de Gardelle V, Yeung N (2017). The impact of evidence reliability on sensitivity and bias in decision confidence. J. Exp. Psychol. Hum. Percept. Perform..

[35] Macmillan, N. & Creelman, C. *Detection Theory: A User’s Guide*. (Psychology Press, 2004).

[36] Kepecs A, Uchida N, Zariwala HA, Mainen ZF (2008). Neural correlates, computation and behavioural impact of decision confidence. Nature.

[37] Boldt A, Yeung N (2015). Shared Neural Markers of Decision Confidence and Error Detection. J. Neurosci..

[38] Calderon CB, Gevers W, Verguts T (2018). The Unfolding Action Model of Initiation Times, Movement Times, and Movement Paths. Psychol. Rev..

[39] Pleskac TJ, Busemeyer JR (2010). Two-stage dynamic signal detection: A theory of choice, decision time, and confidence. Psychol. Rev..

[40] Charles L, King J-R, Dehaene S (2014). Decoding the dynamics of action, intention, and error detection for conscious and subliminal stimuli. J. Neurosci..

[41] Zylberberg A, Barttfeld P, Sigman M, Pereira A (2012). The construction of confidence in a perceptual decision. Front. int. neurosci..

[42] Maniscalco, B., Peters, M. A. K. & Lau, H. Heuristic use of perceptual evidence leads to dissociation between performance and metacognitive sensitivity. *Atten Percept Psychophys* 923–937 (2016). 10.3758/s13414-016-1059-x PMC4811689 26791233

[43] Simon DA, Bjork RA (2001). Metacognition in Motor Learning. J. Exp. Psychol. Learn. Mem. Cogn..

[44] Kilavik BE, Zaepffel M, Brovelli A, MacKay WA, Riehle A (2013). The ups and downs of beta oscillations in sensorimotor cortex. Exp. Neurol..

[45] Gilbertson T (2005). Existing Motor State Is Favored at the Expense of New Movement during 13-35 Hz Oscillatory Synchrony in the Human Corticospinal System. J. Neurosci..

[46] Koelewijn T, van Schie HT, Bekkering H, Oostenveld R, Jensen O (2008). Motor-cortical beta oscillations are modulated by correctness of observed action. Neuroimage.

[47] Swann N (2009). Intracranial EEG reveals a time- and frequency-specific role for the right inferior frontal gyrus and primary motor cortex in stopping initiated responses. J. Neurosci..

[48] Piantoni G, Kline KA, Eagleman DM (2010). Beta oscillations correlate with the probability of perceiving rivalrous visual stimuli. J. Vis..

[49] Bastos AM (2015). Visual Areas Exert Feedforward and Feedback Influences through Distinct Frequency Channels. Neuron.

[50] Siegel M, Warden MR, Miller EK (2009). Phase-dependent neuronal coding of objects in short-term memory. Proc. Natl. Acad. Sci. USA.

[51] Hanslmayr S, Staresina BP, Bowman H (2016). Oscillations and Episodic Memory: Addressing the Synchronization/Desynchronization Conundrum. Trends Neurosci..

[52] Wyart V, Myers NE, Summerfield C (2015). Neural Mechanisms of Human Perceptual Choice Under Focused and Divided Attention. J. Neurosci..

[53] Benchenane K, Tiesinga PH, Battaglia FP (2011). Oscillations in the prefrontal cortex: a gateway to memory and attention. Curr. Opin. Neurobiol..

[54] Thompson E, Varela FJ (2001). Radical embodiment: neural dynamics and consciousness. Trends Cogn. Sci..

[55] Tsuchiya N, Wilke M, Frässle S, Lamme VAF (2015). No-Report Paradigms: Extracting the True Neural Correlates of Consciousness. Trends Cogn. Sci..

[56] Fleming SM, Dolan RJ (2012). The neural basis of metacognitive ability. Philos. Trans. R. Soc. Lond. B. Biol. Sci..

[57] Murphy, P. R., Robertson, I. H., Harty, S. & O’Connell, R. G. Neural evidence accumulation persists after choice to inform metacognitive judgments. *Elife* **4** (2015). 10.7554/eLife.11946 PMC4749550 26687008

[58] Fleming SM, Huijgen J, Dolan RJ (2012). Prefrontal Contributions to Metacognition in Perceptual Decision Making. J. Neurosci..

[59] Desender K, Van Opstal F, Van den Bussche E (2014). Feeling the conflict: the crucial role of conflict experience in adaptation. Psychol. Sci..

[60] Questienne L, Opstal FV, Dijck JV (2016). Metacognition and cognitive control: behavioural adaptation requires conflict experience. Q. J. Exp. Psychol..

[61] Susser JA, Mulligan NW (2015). The effect of motoric fluency on metamemory. Psychon. Bull. Rev..

[62] Hagura, N., Haggard, P. & City, S. Perceptual decisions are biased by the cost to act. *Elife*, 1–20 (2017). 10.7554/eLife.18422 PMC5319835 28219479

[63] Desender K, Calderon CB, Van Opstal F, Van den Bussche E (2017). Avoiding the conflict: Metacognitive awareness drives the selection of low-demand contexts. J. Exp. Psychol. Hum. Percept. Perform..

[64] Pacherie E (2008). The phenomenology of action: A conceptual framework. Cognition.

[65] Lange FPD, Rahnev DA, Donner TH, Lau H (2013). Prestimulus Oscillatory Activity over Motor Cortex Reflects Perceptual Expectations. J. Neurosci..

[66] Song J, Nakayama K (2009). Hidden cognitive states revealed in choice reaching tasks. Trends Cogn. Sci..

[67] Fleming SM (2014). Action-Specific Disruption of Perceptual Confidence. Psychol. Sci..

[68] Pannu JK, Kaszniak AW (2005). Metamemory Experiments in Neurological Populations: A Review. Neuropsychol. Rev..

[69] Rounis E, Maniscalco B, Rothwell JC, Passingham RE, Lau H (2010). Theta-burst transcranial magnetic stimulation to the prefrontal cortex impairs metacognitive visual awareness. Cogn. Neurosci..

[70] Ryals AJ, Rogers LM, Gross EZ, Polnaszek KL, Voss JL (2016). Associative Recognition Memory Awareness Improved by Theta-Burst Stimulation of Frontopolar Cortex. Cereb. Cortex.

[71] Shekhar M, Rahnev D (2018). Distinguishing the Roles of Dorsolateral and Anterior PFC in Visual Metacognition. J. Neurosci..

[72] Bor D, Schwartzman DJ, Barrett AB, Seth AK (2017). Theta-burst transcranial magnetic stimulation to the prefrontal or parietal cortex does not impair metacognitive visual awareness. PLoS One.

[73] Ruby E, Maniscalco B, Peters MAK (2018). On a ‘failed’ attempt to manipulate visual metacognition with transcranial magnetic stimulation to prefrontal cortex. Conscious. Cogn..

[74] Falkenstein M, Hohnsbein J, Hoormann J, Blanke L (1991). Effects of crossmodal divided attention on late ERP components. II. Error processing in choice reaction tasks. Electroencephalogr. Clin. Neurophysiol..

[75] Bates AT, Kiehl KA, Laurens KR, Liddle PF (2009). Low-frequency EEG oscillations associated with information processing in schizophrenia. Schizophr. Res..

[76] Cohen MX, Ridderinkhof KR, Haupt S, Elger CE, Fell J (2008). Medial frontal cortex and response conflict: Evidence from human intracranial EEG and medial frontal cortex lesion. Brain Res..

[77] Cohen MX, Cavanagh JF (2011). Single-Trial Regression Elucidates the Role of Prefrontal Theta Oscillations in Response Conflict. Front. Psychol..

[78] Cavanagh JF, Cohen MX, Allen JJB (2009). Prelude to and Resolution of an Error: EEG Phase Synchrony Reveals Cognitive Control Dynamics during Action Monitoring. J. Neurosci..

[79] Luu P, Tucker DM (2001). Regulating action: alternating activation of midline frontal and motor cortical networks. Clin. Neurophysiol..

[80] Jensen O, Lisman JE (2000). Position Reconstruction From an Ensemble of Hippocampal Place Cells: Contribution of Theta Phase Coding. J. Neurophysiol..

[81] Cavanagh JF, Frank MJ (2014). Frontal theta as a mechanism for cognitive control. Trends Cogn. Sci..

[82] Dragoi G, Buzsáki G (2006). Temporal Encoding of Place Sequences by Hippocampal Cell Assemblies. Neuron.

[83] Sauseng P (2006). Relevance of EEG alpha and theta oscillations during task switching. Exp. Brain Res..

[84] van Driel J, Sligte IG, Linders J, Elport D, Cohen MX (2015). Frequency Band-Specific Electrical Brain Stimulation Modulates Cognitive Control Processes. PLoS One.

[85] van de Vijver I, Ridderinkhof KR, Cohen MX (2011). Frontal Oscillatory Dynamics Predict Feedback Learning and Action Adjustment. J. Cogn. Neurosci..

[86] Fleming SM (2016). Changing our minds about changes of mind. Elife.

[87] Holroyd CB, Coles MGH (2002). The neural basis of human error processing: Reinforcement learning, dopamine, and the error-related negativity. Psychol. Rev..

[88] Cleeremans A (2011). The Radical Plasticity Thesis: How the Brain Learns to be Conscious. Front. Psychol..

[89] Cleeremans A, Timmermans B, Pasquali A (2007). Consciousness and metarepresentation: a computational sketch. Neural Netw..

[90] Buzsaki, G., Peyrache, A. & Kubie, J. Emergence of Cognition from Action. *Cold Spring Harb. Symp. Quant. Biol*. (2014). 10.1101/sqb.2014.79.024679 PMC4895837 25752314

[91] Buzsaki, G. *The Brain from Inside Out*. (Oxford University Press, USA., 2019).

[92] Benwell, C. S. Y., Beyer, R., Wallington, F. & Ince, R. A. A. History biases reveal novel dissociations between perceptual and metacognitive decision-making. *bioRxiv Prepr* (2019). 10.1167/jov.23.5.14 PMC10207958 37200046

[93] Rouault, M., Dayan, P. & Fleming, S. M. Forming global estimates of self-performance from local confidence. *Nat. Commun*. 1–11 (2019). 10.1038/s41467-019-09075-3 PMC6408496 30850612

[94] Wilson RC, Takahashi YK, Schoenbaum G, Niv Y (2014). Orbitofrontal cortex as a cognitive map of task space. Neuron.

[95] Schuck, N. W., Wilson, R. & Niv, Y. In *Goal-Directed Decision Making* 259–278 (Elsevier Inc., 2018).

[96] Schuck NW, Cai MB, Wilson RC, Niv Y, Road W (2016). Human Orbitofrontal Cortex Represents a Cognitive Map of State Space. Neuron.

[97] Wokke ME, Knot SL, Fouad A, Richard Ridderinkhof K (2016). Conflict in the kitchen: Contextual modulation of responsiveness to affordances. Conscious. Cogn..

[98] Wokke, M. E. & Ro, T. Competitive Frontoparietal Interactions Mediate Implicit Inferences. SO – J. Neurosci. 2019 Jun 26;39(26):5183–5194. *J. Neurosci* (2019). 10.1523/JNEUROSCI.2551-18.2019 PMC6595957 31015338

[99] Maniscalco B, Lau H (2012). A signal detection theoretic approach for estimating metacognitive sensitivity from confidence ratings. Conscious. Cogn..

[100] Vigário RN (1997). Extraction of ocular artefacts from EEG using independent component analysis. Electroencephalogr. Clin. Neurophysiol..

[101] Mitra PP, Pesaran B (1999). Analysis of Dynamic Brain Imaging Data. Biophys. J..

[102] Cohen, M. X. Comparison of different spatial transformations applied to EEG data: A case study of error processing. *Int. J. Psychophysiol*. (2015). 10.1016/j.ijpsycho.2014.09.013 25455427

[103] Cohen, M. X. Analyzing Neural Time Series Data: Theory and Practice. *MIT Press* (2014).

[104] Oostenveld R, Fries P, Maris E, Schoffelen J-M (2011). FieldTrip: Open Source Software for Advanced Analysis of MEG, EEG, and Invasive Electrophysiological Data. Comput. Intell. Neurosci..

